# Phage therapy of wound-associated infections

**DOI:** 10.1007/s12223-021-00946-1

**Published:** 2022-01-13

**Authors:** Anna Zyman, Andrzej Górski, Ryszard Międzybrodzki

**Affiliations:** 1 Pharmacology Undergraduate Programme, School of Physiology, Pharmacology and Neuroscience, Faculty of Life Sciences, University of Bristol, University Walk, Bristol, BS8 1TD UK; 2 Bacteriophage Laboratory, Ludwik Hirszfeld Institute of Immunology and Experimental Therapy, Polish Academy of Sciences, 53-114 Wrocław, Poland; 3 Phage Therapy Unit, Ludwik Hirszfeld Institute of Immunology and Experimental Therapy, Polish Academy of Sciences, 53-114 Wrocław, Poland; 4 Infant Jesus Hospital, Medical University of Warsaw, 02-005 Warsaw, Poland; 5 Department of Clinical Immunology, Transplantation Institute, Medical University of Warsaw, 02-006 Warsaw, Poland

## Abstract

Phages are viruses which can specifically infect bacteria, resulting in their destruction. Bacterial infections are a common complication of wound healing, and experimental evidence from animal models demonstrates promising potential for phage-dependent eradication of wound-associated infections. The studies discussed suggest that phage therapy may be an effective treatment, with important advantages over some current antibacterial treatments. Phage cocktails, as well as co-administration of phages and antibiotics, have been reported to minimise bacterial resistance. Further, phage-antibiotic synergism has been reported in some studies. The ideal dose of phages is still subject to debate, with evidence for both high and low doses to yield therapeutic effects. Novel delivery methods, such as hydrogels, are being explored for their advantages in topical wound healing. There are more and more Good Manufacturing Practice facilities dedicated to manufacturing phage products and phage therapy units across the world, showing the changing perception of phages which is occurring. However, further research is needed to secure the place of phages in modern medicine, with some scientists calling upon the World Health Organisation to help promote phage therapy.

## Introduction

Considered the most abundant biological entities in the biosphere (Kifelew et al. 2020), it is estimated that there are 10^31^ naturally occurring phages in the world (Macdonald et al. 2020). Shortly after bacteriophages (phages) were independently discovered by Twort in 1915 and by d’Hérelle in 1917, phages were applied widely as antibacterial agents (Chanishvili 2012). Upon Fleming’s discovery of antibiotics in 1928, phages were largely forgotten in the Western world due to the efficacy and promise held by antibiotics. However, nowadays, multidrug resistant (MDR) bacteria are becoming a major problem. A recent study commissioned by the UK government estimates that by 2050, the annual number of people dying from antimicrobial resistance could reach 10 million (O’Neill 2014), outnumbering current yearly cancer deaths. Table 1 presents the most dangerous antibiotic-resistant pathogens according to the World Health Organisation (WHO). The threat this poses to global health has stimulated research in alternative therapies, reviving interest in phages.

**Table 1:** Priority list for development of new antibiotics according to the World Health Organisation

**Priority**	**Pathogen species**	**Antimicrobial resistance**
Critical	*Acinetobacter baumannii*	Carbapenem-resistant
	*Pseudomonas aeruginosa*	Carbapenem-resistant
	Enterobacteriaceae*: (*Enterobacteriaceae include: *Klebsiella pneumonia*, *Escherichia coli*, *Enterobacter* spp., *Serratia* spp., *Proteus* spp. and *Providencia* spp., *Morganella* spp.)	Carbapenem-resistant, 3rd generation cephalosporin-resistant
High	*Enterococcus faecium*	Vancomycin-resistant
	*Staphylococcus aureus*	Methicillin-resistant, vancomycin intermediate and resistant
	*Helicobacter pylori*	Clarithromycin-resistant
	*Campylobacter*	Fluoroquinolone-resistant
	*Salmonella* spp.	Fluoroquinolone-resistant
	*Neisseria gonorrhoeae*	3rd generation cephalosporin-resistant, fluoroquinolone-resistant
Medium	*Streptococcus pneumoniae*	Penicillin-non-susceptible
	*Haemophilus influenzae*	Ampicillin-resistant
	*Shigella* spp.	Fluoroquinolone-resistant

Adapted from (Tacconelli and Margrini 2017)

Bacterial infections are a common and problematic complication of wound healing. Bacterial contamination occurs in all wounds and is caused by environmental factors, nearby skin and endogenous patient sources (Siddiqui and Bernstein 2010). However, this contamination does not always progress to colonisation and infection. The main bacteria responsible for infection in acute and chronic wounds are *Staphylococcus aureus* and *Pseudomonas aeruginosa* (Bowler et al. 2001). Furthermore, *P. aeruginosa* and *Escherichia coli* are some of the most prevalent bacteria known to colonise infected wounds (Oliveira et al. 2018). Taking the example of chronic venous leg ulcers, it has been reported that the most common bacteria found in this type of wound include *S. aureus*, *E. faecalis*, *P. aeruginosa*, coagulase-negative staphylococci and *Proteus* spp. (Siddiqui and Bernstein 2010). Many of the aforementioned bacteria appear on the WHO’s list of priority pathogens for the research and development of new antibiotics (Table 1), thus highlighting the importance of new antibacterial treatments for wound-associated infections. Using phage therapy to eradicate wound-associated infections shows interesting therapeutic potential for clinical applications. This paper aims to review the efficacy of phage therapy for infected wounds, highlighting the most recent experimental evidence supporting phage therapy for infected wounds, novel delivery methods and co-administration of phages and antibiotics.

## Experimental evidence supporting the efficacy of phage therapy for the treatment of infected wounds

There is plenty of evidence that phages are beneficial in treating wound-associated infections, and phage therapy should generally be considered highly effective when administered to patients appropriately (Steele et al. 2020). Some early studies, such as a study by Soothill in 1994, investigated the role phages could play in preventing infections from occurring in skin grafting procedures. The wounds inflicted on the guinea pig models were infected with *P. aeruginosa* and/or phage and/or control suspension. *P. aeruginosa* frequently colonises burns, resulting in skin graft failure. In this study, phage BS24 was applied to wounds before grafting to evaluate whether it could prevent infection. It was demonstrated that phage BS24 protected grafts against infection with *P. aeruginosa* (as seen by graft survival), thus confirming that phages can be beneficial in preventing wound-associated infections (Soothill 1994).

Despite clear indication of the possible efficacy of phage therapy in the treatment of infected wounds, there is conflicting evidence in the literature concerning the ideal dose of phages that should be administered to yield the best therapeutic effects. Some studies demonstrate that low concentrations of phages result in better effects. For example, a study by Shetru et al. (2021) found that mice treated with a lower dose of ΦDMSA-2 phages (5 × 10^6^ plaque-forming units) in vivo in an excisional wound infected with *S. aureus* resulted in faster wound healing than a higher phage dose (10^7^ PFU). Indeed, bacteria were cleared by day 16 with low phage dose, whereas they could still be found in the wound treated with a higher dose of phages on that day. In contrast, a study by Mendes et al. (2013) carried out on diabetic foot infections in rodent and porcine models used high doses of phages to treat the infections. It is not possible to draw a classic concentration–response curve for phages due to their replication at the site of the wound. Some suggest that an ideal concentration for topical phage application could be between 10^6^ and 10^8^ PFU/mL (Steele et al. 2020). Chang et al. (2020) advocate for higher phage concentrations, recommending 10^8^–10^9^ PFU/mL for biocontrol of bacteria. It is generally believed that phages should reach sufficient densities (titres) to be effective antibacterial agents (Danis-Wlodarczyk et al. 2021). In fact, rapid drop of phage titres has probably been responsible for the failure of the PhagoBurn clinical trial of phage effectiveness in wound infections (Jault et al. 2019). It has to be kept in mind that — contrary to other medicines — high phage titres at sites of infections can be reached not only by massive phage administration but also by local phage multiplication on invading bacteria. Evidently, the latter option seems to be more compatible with effective therapy. Therefore, administering high phage doses may not always translate into therapeutic success — especially that higher phage dosage may lead to enhanced production of phage-neutralising antibodies which could interfere with the therapy outcome (Lusiak-Szelachowska et al. 2017; Zaczek et al. 2019b).

Thus, both high and low doses of phages in the treatment of infected wounds have proven to be effective but further research in this area appears necessary.

Interestingly, in the study by Mendes et al. (2013), it was found that the epithelial gap was reduced in wounds treated by phages which were infected by *S. aureus* and *P. aeruginosa*, and that phages for *A. baumannii* were able to induce a bacterial reduction at the site of the wound. Furthermore, this study found evidence that phages can promote wound healing in addition to simply combatting the infection.

Diabetic foot infections are a serious complication for patients suffering from diabetes mellitus and are currently treated by debridement and antibiotics (Mendes et al. 2013), which aim to prevent and cure infection. A recent in vivo study by Kifelew et al. (2020) in diabetic mouse wound infections investigated the efficacy of AB-SA01 (a cocktail of 3 *Myoviridae* phages of *S. aureus*). This study compared vancomycin and phage-treated mice and found that wound healing was similar for both treatment options. However, it was also found that the decrease in bacterial load caused by AB-SA01 was of equal or greater efficacy than in vancomycin-treated mice. From this study, it is not unreasonable to consider that phages could be a viable alternative to antibiotic treatment provided this finding translates to human models. This study makes an important point about the models we use for studying phages, as skin wound healing is different in rodents and humans. Rodents have an additional step in their wound healing process called skin contraction. This difference was overcome in the study by using a splint wound model thus preventing the wounds from contracting and rendering the model more human-like. The safety and tolerability of AB-SA01 was evaluated in a clinical trial for topical administration, yielding positive results as patients received it well (Ooi et al. 2019).

Considering that phage therapy is a relatively unheard-of treatment option in the Western world, it is interesting to know how it is perceived by patients who may need it. One such survey was carried out by Macdonald et al. in 2020 to evaluate how patients perceived phage therapy for diabetic foot infections. It found that 86.8% of the participants were willing to undergo phage therapy if recommended by their doctor, and some of the remaining participants indicated needing more information as one of the reasons for their reluctance. This high endorsement of phage therapy should be interpreted with caution, as the results may not be fully representative. The study only counted 55 participants and relied on a self-selection process, meaning that motivated individuals were more likely to take part. Nevertheless, this study shows willingness from patients to undergo phage therapy or to learn more about it.

Phage cocktails can target a broader range of strain-specific bacteria (Pinto et al. 2020) and are an important strategy for minimising resistance developing in bacteria (Steele et al. 2020). Indeed, Pinto et al. (2020) report the emergence of several resistant bacterial strains after only a few hours of monophage therapy. Phage cocktails reduce the number of phage-resistant bacterial mutants and increase the speed at which bacteria are killed compared to monophage therapy (Pinto et al. 2020). Additionally, phage cocktails were found to produce significant reduction in wound bioburden, faster tissue healing and greater wound contraction in a study carried out in mice (Chhibber et al. 2018). Furthermore, better phage persistence at the wound site has been linked to liposomal entrapment of the phage cocktail, as supported by in vitro stability studies and in vivo phage titre determination (Chhibber et al. 2018).

These are only a few of the many studies demonstrating the efficacy of phages in treating infected wounds in animals. Thus, there is clear experimental evidence of the therapeutic effects of phages, and future studies should focus on more specific aspects of phages, such as how to fine-tune their characteristics for the best possible therapeutic outcomes (Pirnay et al. 2021) and their translatability to human models. This is well illustrated by the results of PhagoBurn phase 1/2 randomised, placebo-controlled trial which aimed to evaluate the efficacy and safety of phages in the treatment of burn wounds infected by *E. coli* and *P. aeruginosa* (Jault et al. 2019). Due to the lack of stability of the phage cocktail used, which resulted in the application of a much lower phage dose than expected and the problem recruiting patients fulfilling qualification criteria, its only positive result was the observation of less incidence of adverse events in the small (*n* = 13) phage-treated group than in controls. Unfortunately, the antibacterial efficacy of phage cocktail PP1131 was inferior to the current standard of care. Hopefully, further clinical studies, case study reports and systematic reviews will shed much more positive light on the results of phage use in the treatment of infected wounds, especially in diabetic patients (Fish et al. 2016; Gupta et al. 2019; Nadareishvili et al. 2020; Duplessis and Biswas 2020). Table 2 summarises the key findings on the effectiveness of phage therapy based on experimental evidence.

**Table 2:** Overview of the key findings on the efficacy of phage therapy for the treatment of infected wounds based on experimental evidence

**Observation/interpretation**	**Model used**	**Experimental techniques used**	**Phage used**	**Type of article and reference**
Phage therapy could play a role in preventing infections from occurring in skin grafting procedures	Guinea pig (in vivo)	Skin graft procedure to represent a burn model	BS24	Research article (Soothill 1994)
Evidence that phages can promote wound healing in addition to simply combatting infection	- Wistar rats (in vivo) - Yorkshire pigs (in vivo)	Induction of diabetes mellitus in the animals, infliction of wounds, inoculation of wounds, followed by debridement and phage treatment. Swabs were collected and wounds photographed with a digital microscope	Cocktails used: - *S. aureus* F44/10 and F125/10 - *P. aeruginosa* F770/05 and F510/08 - *A. baumannii* F1245/05	Research article (Mendes et al. 2013)
Viable alternative to antibiotics: decrease in bacterial load caused by AB-SA01 was of equal or greater efficacy than in vancomycin treated mice	Balb/c mice (in vivo)	Induction of diabetes mellitus in the animals, infliction of excisional wounds, followed by phage or vancomycin treatment. Swabs were collected and wound sizes measured	AB-SA01 (cocktail of 3 *S. aureus Myoviridae* phages)	Research article (Kifelew et al. 2020)
Positively perceived	Humans	Survey distributed to eligible patients in Scotland	Not applicable	Research article (Macdonald et al. 2020)
Phage cocktails reduce the number of phage-resistant bacterial mutants as well as increase the speed at which bacteria are killed compared to monophage therapy	Not applicable as this observation comes from a review, not a research article	Not applicable as this observation comes from a review, not a research article	Some examples: - 14/1, PNM and ISP - vB_EcoS_CEB_EC3a and vB_PaeP_PAO1-D	Review (Pinto et al. 2020)
Phage cocktails produce significant reduction in wound bioburden, faster tissue healing and greater wound contraction in a study carried out in mice	BALB/c mice (in vivo)	Induction of diabetes mellitus in the animals, infliction of wounds, inoculation of wounds, followed by local administration of monophage therapy or a phage cocktail Wound bioburden and phage titre were monitored	Cocktail of *S. aureus* MR-5 and MR-10	Research article (Chhibber et al. 2018)
PhagoBurn: less incidence of adverse events in the small (*n* = 13) phage-treated group than in controls	Humans	Phase 1/2 trial comparing treatment with PP1131 and the standard of care in patients with *P. aeruginosa* burn wound infections (taking daily swabs for analysis)	PP1131 (phage cocktail made of 12 anti *P. aeruginosa* phages)	Article (Jault et al. 2019)
Bacterial resistance to phage therapy is often due to mutations in bacterial receptors for phages, such as lipopolysaccharide (LPS) and type IV pili (T4P)	Not applicable as this observation comes from a review, not a research article	Not applicable as this observation comes from a review, not a research article	Not applicable as no specific examples were given in the review	Review (Vaitekenas et al. 2021)
Call for caution in the development of phage cocktails as not all cocktails result in enhanced cell killing	No animal model was used in this study	Characterisation of 5 phages: - Transmission electron microscopy - Genome sequencing - Time-kill experiments	- SPCB - SPCG - SMS12 - SMS21 - SMS29	Article (Pinto et al. 2021)
Biofilm reduction after treatment with phage	Not applicable	High content screening assay, use of endotracheal tube	- vB_PaeM_USP_2 - vB_PaeM_USP_18	Article (Oliveira et al. 2021)
PA4 could cause significant reduction of in vitro bacterial growth	Not applicable	- Planktonic adsorption and inhibition assays - Crystal violet biofilm assay - Live/dead staining - Genome sequencing	PA4	Article (Camens et al. 2021)

## Administering phages to infected wounds

### Novel phage delivery methods (focus on hydrogels)

Research is currently exploring different ways to administer phages locally to infected wounds. Several options are available for topical application, including gels, creams and ointments. Here, attention will be particularly devoted to hydrogels, one of the phage delivery methods currently in vogue (Pinto et al. 2020). Wound dressings are a beneficial option for topical treatment of wounds as they keep adequate concentrations of the treatment at the site of infection, preventing wound exudate from washing it away (Shen et al. 2021).

There is increasing evidence from preclinical studies (in vitro and in vivo) suggesting that hydrogels are an effective way of administering phage therapy to patients (Kim et al. 2021). As some phages inactivate in alcohol, hydrogels are a very suitable way of delivering phages due to their hydrophilic properties (Chang et al. 2020). Furthermore, hydrogels improve the hydration balance in an infected wound and allow hydrogen bonds to form between the water and phage proteins, which is an important factor for the stabilisation of the phages (Chang et al. 2020). A recent study investigated the properties and antibacterial activity of hydrogel fibres. The hydrogel fibres were embedded with phages and used as a wound dressing (Shen et al. 2021). This study successfully demonstrated that phage-embedded hydrogel fibres can slowly release lytic phages which are active at the site of infection and can stop bacterial growth for up to 24 h. The proportion of phages remaining lytic once embedded in the hydrogel samples was 80%. Importantly, phages did not affect the tensile properties of the fibre, and the fibre did not affect phages’ bactericidal properties. Indeed, phage tails need to be free for the phages to exert their effects properly. Using a phage-modified surface jeopardises this which is why phages were encapsulated in a hydrogel matrix, thus overcoming the potential challenge of immobilisation (Shen et al. 2021). Another study investigated thermosensitive hydrogel wound dressings, a solution which becomes semi-solid at skin temperature and takes the shape of the wound (Yan et al. 2021). This allows for drug depot at the site of injury and prevents further damage from being inflicted on the wound. Thermosensitive wound dressings allow for sustained release of phages at the site of the wound, ensuring enough phage product is present at the site of infection and improving antibacterial activity compared to applying free phage suspension. This study found that including phages in a hydrogel did not affect phage’s bactericidal properties in any significant way and that thermosensitive hydrogel wound dressings containing phages can target biofilms, thus making them a good therapeutic option for chronic wound infections.

Concerning the stability and safety profile of hydrogels, a recent review by Kim et al. (2021) highlights the main points of interest in this area. Phage stability depends on several factors, namely temperature, pH, light exposure and formulation composition. Whenever possible, this study suggests incorporating phages after formulation of the hydrogel, thus increasing their probability of stabilising. Although phage-free hydrogels have been studied more than phage-embedded hydrogels, cytocompatibility, cell cytotoxicity and hemocompatibility are listed as some of the techniques used to establish the safety profile of phage hydrogels (Kim et al. 2021). To date, there is only preliminary evidence that phage-embedded hydrogels are safe for topical application on skin or subcutaneous injections and the long-term effects of exposure to phage hydrogels are unknown.

Creams are another delivery method of phage therapy. Chang et al. (2020) refer to a phage cream which displayed clear antibacterial activity, but had an unknown phage-release profile. Non-ionic-based creams are recommended for topical application of phages (Chang et al. 2020). Although immobilised phages on wound dressings are an interesting concept, difficulties may be encountered in its shelf-life, which would need to be tested and optimised to render it a viable treatment option (Chang et al. 2020).

Thus, novel delivery methods for phages carry a lot of promise for the future of wound care and confirm in a more practical scenario the role of phages in the eradication of wound-associated infections.

### Combination therapies: phages and antibiotics

In addition to administering phages alone, it is also possible to administer them in conjunction with other treatments. A common treatment co-administered with phages is antibiotics, reported to exert a synergistic effect in some studies, such as Engeman et al. (2021). This study on multidrug-resistant *P. aeruginosa* infections found that antibiotics and phages have a synergistic effect in reducing bacterial population (Engeman et al. 2021), whilst also re-sensitising *P. aeruginosa* to antibiotics. Moreover, results of this study suggest that combination treatment is not only beneficial to antibiotic treatment, but also to phage therapy as a decreased amount of phage resistant bacteria was reported. It is also suggested that bacteria can only resist the combination treatment by becoming avirulent, which would enable the infection to be cleared by the host’s immune system and thus still achieve the desired outcome of curing the patient. Phage-antibiotic synergy is thought to be caused by a mechanism whereby bacteria elongate at a cellular level due to the action of the antibiotics, promoting the replication of phages and possibly external attachment to the bacteria as it exhibits a larger surface area (Huon et al. 2020). Furthermore, phage-antibiotic synergy seems to work better when phages are administered before antibiotics (Pirnay et al. 2021). Some studies, such as a study by Huon et al. (2020), find that there is no synergism between phages and antibiotics. Indeed, this study found that co-administration of phages and antibiotics did not bring better results than administration of phages alone. It is suggested by some that antibiotic class is an important factor influencing phage-antibiotic combination (Gu Liu et al. 2020).

Concerning the issue of bacterial resistance to treatments which is highly prevalent against some antibiotics, several comments can be made. Firstly, there is potential for cross-resistance to occur simultaneously to phages and antibiotics, which should be considered when co-administering these two treatments (Pirnay et al. 2021). However, unlike antibiotics which are static, phages display an ability to overcome bacterial resistance (Pirnay et al. 2021), although these mechanisms are currently poorly understood (Taati Moghadam et al. 2020). Some recent evidence suggests that phages mutate and diversify to overcome bacterial resistance, without acquiring undesirable genes (Botka et al. 2019). Stronger resistance tends to develop toward co-administration of the same type of antibacterial agent, such as phage-phage or antibiotic-antibiotic (Pinto et al. 2020). From a practical point of view, it is also far easier to develop a new phage than a new antibiotic (Shetru et al. 2021). Some defence mechanisms developed by bacteria to resist phage therapy have been suggested in the literature, including bacteria cleaving phage DNA through restriction-modification systems and blocking phage DNA replication (Taati Moghadam et al. 2020).

More advantages to phage therapy compared to a course of antibiotics have been reported in the literature. A research article by Kifelew et al. (2020) states that bacterial load continues to decrease after treatment with phages, whereas bacterial load remains constant after antibiotic treatment. Another key advantage phages have over antibiotics is that they do not disrupt the intestinal microbiota (Huon et al. 2020). Furthermore, phages have lower development costs, are relatively free of side effects and reduce the potential for overgrowth of a secondary pathogen, thus lowering the chances of a secondary treatment (Taati Moghadam et al. 2020). Disadvantages of phages over antibiotics are that phages will not replicate if their target organism is not present, and the host immune system can recognise them as foreign invaders and clear them from systemic circulation (Taati Moghadam et al. 2020).

A different treatment co-administered with phages is honey, evaluated in a study by Oliveira et al. (2018). This study was able to demonstrate that treatment by both honey and phages on dual-species biofilms on polystyrene resulted in enhanced killing activity compared to treatment with either therapeutic agent alone. However, this improvement was not classified as synergistic by the authors as it was only minor (Oliveira et al. 2018).

Thus, co-administering phages with antibiotics seems a promising option for the eradication of wound-associated infections, although neither the exact mechanisms at play nor the intricacies of this interaction are fully understood, and further studies are required.

## Future perspectives

The data derived from experiments in animals as well as from clinical studies indicate that phage therapy of wound infections may offer a promising solution at a time when antimicrobial resistance increasingly threatens global health. The setting of chronic venus ulcers and diabetic wound infections seem to be the most challenging dilemmas of therapy and therefore are the most obvious target for studies aiming at confirming its value. Clearly, more evidence from human studies is needed to ascertain the true value of the therapy. This is especially relevant in light of the data indicating that wound healing is different in rodents and humans. Furthermore, formal clinical trials carried out according to the current standards of Evidence-Based Medicine are urgently needed to determine the position of phage therapy in the modern treatment of contemporary medicine. Moreover, the findings showing that phages may directly promote wound healing seem to be particularly interesting. Recent data indicate that phages may target not only bacteria but also eukaryotic cells and penetrate/trancytose those cells (Zaczek et al. 2019a). Furthermore, epithelial cells penetrated by phages may be protected from pathogen-induced injury and apoptosis (Gorski et al. 2020). Further studies on phage-epithelial cell interactions may yield novel data shedding more light on those phage-mediated effects unrelated to their anti-bacterial action with potential application in wound healing.

In addition to carrying out more studies, it will also be important to work towards creating a legal framework for the use of phage therapy. Currently, only Georgia and Russia allow phages to be used as standard medical application (Pinto et al. 2020), and some other countries allow phages to be used as compassionate therapy under article 37 of the Helsinki Declaration. Pinto et al. (2020) highlight the value of phage therapy becoming a standard medical practice legally. Hopefully, this can take place soon, as several recent reviews are clear in their claim and provide substantial evidence that phage therapy is safe (Steele et al. 2020; Duplessis and Biswas 2020).

Precision medicine is a topical subject in modern medicine, and phage therapy lends itself particularly well to this model as it has the potential to generate bespoke treatments for each wound-associated infection (Steele et al. 2020). Although this is a possible way to further develop phage therapy, it is suggested that fixed cocktails may also have a role to play in initial clinical application while waiting for confirmation of in vitro lytic activity (Duplessis and Biswas 2020).

## Conclusion

Abundant evidence from the literature supports phage therapy for the treatment of wound-associated infections, and some suggest that phages might have a role in actively promoting wound healing. Many studies have reported high efficacy of phage therapy in animal models, and future studies should focus on translating these results to humans and carrying out more clinical trials. In a world where bacterial resistance is threatening global health, phages provide several paths to minimise the development of bacterial resistance, such as using phage cocktails instead of monophage therapy, or antibiotic and phage co-administration. Novel delivery methods, like hydrogels, are being explored as new ways to administer phages to fine-tune their characteristics for the best possible therapeutic outcomes on infected wounds.

It is important to recognise the change in perception of phages which is occurring. There are more and more Good Manufacturing Practice facilities dedicated to the development of phage products in the world (Chang et al. 2020) and more and more phage therapy units, in countries such as Georgia, Poland, USA, Australia and Belgium (Knezevic et al. 2021). Many scientists call upon the WHO to help develop phage therapy, by helping to promote its knowledge worldwide and build a “regulatory system for phage products through its vaccines prequalification (PQ) program” (Knezevic et al. 2021).
